# Commentary: Physical time within human time

**DOI:** 10.3389/fpsyg.2023.1092351

**Published:** 2023-06-07

**Authors:** Gustavo E. Romero

**Affiliations:** ^1^Instituto Argentino de Radioastronomía (IAR), Consejo Nacional de Investigaciones Científicas y Técnicas (CONICET), Comisión de Investigaciones Científicas de la Provincia de Buenos Aires (CICPBA), Universidad Nacional de La Plata (UNLP), Buenos Aires, Argentina; ^2^Facultad de Ciencias Astronómicas y Geofísicas, Universidad Nacional de La Plata, Buenos Aires, Argentina

**Keywords:** time, temporal experience, present, spacetime, IGUS, change, temporal order

Time has always been a source of perplexity and fascination for human beings. Presocratic philosophers initiated the first discussions on the reality of time and its relation to change. Heraclitus presumed that change was a basic and irreducible ingredient of nature. According to him, the world would be a manifold of substances in permanent change. Parmenides, on the contrary, famously denied change. He argued, in what was possibly the first deductive argument in the history of ontology, that change is impossible because it demands that what is not, should somehow be. He relentlessly concluded that our image of a dynamic universe is a pure illusion: reality is fixed, coming to be and perishing are excluded from the cosmos, and whatever exists must be permanent (see Graham, 2006 for a fascinating account of Parmenides's challenge).

The discussion between Heraclitus and Parmenides permeates the entire history of Western thought and has ended up reaching our days in the form of a tension between two evidently irreconcilable conceptions of time: the flowing time and spacetime. The idea that time somehow flows is closely related to the idea that there is a specific instant called “the present” that, through change, is sequentially actualized: old instants no longer exist, so there is no past but just our memory of what once was. Future instants do not exist yet. Only the “now” is real and is permanently changing. Such a view is usually called “presentism.”

The opposite view of presentism is “eternalism,” also called the “block universe view.” Present, past, and future moments (and hence events) exist. They form a four-dimensional “block” of spacetime. Events are ordered by relations of earlier than, later than, or simultaneous with, one another. These relations among events are unchanging. They cannot change because time *already is* one of the dimensions of the “block,” and change is a variation with respect to time. It is not correct, however, to infer that this view represents a “static reality.” Worldlines in spacetime describe physical processes, i.e., a series of changes in material things. A change can be defined by an ordered pair of physical states, each at a different time. A physical state is just a collection of the properties of a system at a given time. Therefore, it is said that something changes if, between two different moments *t*_1_ and *t*_2_, any of its properties do not remain identical. Thus, the “block universe” is full of change because the things that make it up are in different states at different times. Of course, what does not change, and cannot change, is the “block universe” itself. How could it change if time is one of its dimensions? To change, the universe would have to be a five-dimensional entity, and two of those dimensions would have to be timelike. Therefore, it might be possible to state that a four-dimensional portion of the block changes with respect to the fifth dimension. Then, the five-dimensional “block” would be fixed, unless there is a sixth time dimension, and so on. However, the world does not seem to be like that: it is four-dimensional, with three spatial and one temporal dimension, and that is it. The change is already within spacetime. That is why general relativity, our best theory of spacetime, is a dynamic theory: It describes how entities in three dimensions can change with respect to a fourth.

Dean Buonomano has recently pointed out in a discussion with Carlo Rovelli that:

“(…) unlike the empirically confirmed predictions of relativity (e.g., clocks slow down at high gravitational potentials), it is important to stress that there is no empirical evidence for the block universe. Indeed, it is far from clear that there are any testable predictions that could prove or disprove the existence of the block universe (other than the emergence of a confirmed time traveler).” (Buonomano and Rovelli, 2021).

I believe that this statement is disputable.^1^ The “block” of the block universe has a geometric structure that is determined through Einstein field equations. According to these equations, any perturbation in the matter will result in a perturbation of spacetime; therefore, there will be an energy flux in the form of gravitational waves across the spacetime manifold. Such a flux can exist through empty spacetime only if its dimensionality is at least 4 (Romero, 2017). This means that, if presentism is correct and the world is essentially three-dimensional, phenomena such as gravity waves should not occur (Romero, 2018). However, the experiment indicates the opposite: gravity perturbations travel from distant sources to the Earth where experiments such as LIGO can detect them. We conclude that the world is four-dimensional and, consequently, past, present, and future exist. There is no need to resort to time travel, although the existence of time travelers is not forbidden by general relativity (as correctly noticed by Rovelli in the same article).

Even if presentism is inconsistent with general relativity, our brain undoubtedly experiences a sense of “newness.” What is the origin of this sense? Earlier, I suggested,

“I maintain that ‘nowness' and ‘hereness' emerge from the existence of perceiving self-conscious beings in a certain environment. What these beings perceive is not time, but changes in things (…). Similarly, they do not perceive space but spatial relations among things. In particular, we do not perceive the passage of time. We perceive how our brain changes. I claim that there is no present *per se*, in the same way that there is no smell, no pain, no joy, no beauty, no noise, and no secondary qualities at all without sentient beings. What we call ‘the present' is not in the world. It emerges from our interaction with the world.” (Romero, 2015).

Perhaps this can be expressed more simply by saying that what we call consciousness arises from groups of successive brain events arranged in some specific configurations in spacetime. Those events are changes and processes, that is, chains of pairs of states that associate properties of one part of the brain with properties of other regions, either in the same brain or in the local environment, at slightly different times. Since these properties are not the same across the time dimension, the illusion of a “time flow” arises. However, time does not flow in any meaningful, non-metaphorical sense. It is just one dimension along which spatial properties vary. The “flow” of time is just a brain construct, an illusion, albeit a very stubborn one because it is rooted in what defines our very identity. The variation, I insist, is only a relative difference in the distribution of properties along the manifold that represents the four-dimensional spacetime.

In their recent article “*Physical time within human time*,” Gruber et al. (2022) reported on a new experimental setting aimed at verifying the hypothesis that the passage of time is a construction of the brain. The basic idea is that the experience of the flow of time is not a representation by a passive recipient of sensory stimulation but is generated by predictive processes of the brain and proactive sensorimotor activity of the whole body. Gruber et al.'s approach consists of enhancing and constructing an “information gathering and utilizing system” (IGUS) capable of manipulating the experience of the past, present, and future. This would allow us to put the hypothesis of a brain-constructed experiential time to the test.

The idea of IGUS was introduced by Hartle (2005) and discussed by Romero (2015) and Huggett (2018) from a philosophical and physical point of view. The practical construction of IGUS presented by Gruber and Smith (2019) opens the door to new laboratory experiments that might allow a thorough investigation of the biological basis of perceptual time.

To succeed in the manipulation of time perception, a specific IGUS should control the information on the environment provided to the processing system (the human brain). This is achieved with the immersion of the subject in a virtual reality fed with a system of cameras whose output is controlled and allows the researcher to switch between present, past, or future moments. The resulting “present” experienced is not unique and hence not a property of spacetime but rather of the specific IGUS. This is a very important result obtained by Gruber et al.: the two diverging ideas of time, the physical, objective time and the human, subjective one, are the result of the same and unique set of physical laws. The neuroscience and physics of time seem to accord through mechanisms that can be objectively tested.

Further experiments should evaluate the efficacy of different IGUS configurations to implement tasks related to the survival skills of the individual. Complexity and a variety of new tests can be obtained by introducing various gadgets. The comparison of the results of such research might lend support to the hypothesis advanced by Hartle (2005) that the IGUS of the human brain is that which is best suited for survival in its environment. The picture emerging from these investigations, to date, seems to tell us that:

“(…) the present does not flow or move. Only material individuals (and their brains, if they have one) can change and move. Becoming is not a property of physical events but of the consciousness of the events. We call ‘becoming' to the series of states of consciousness associated with a certain string of physical changes. Events do not become. Events just *are*.” (Romero, 2015).

## Author contributions

The author confirms being the sole contributor of this work and has approved it for publication.

## Footnotes

^1^I consider the classical general theory of relativity as the correct theory to model reality (and hence of time), at least at the scales relevant to the issues discussed in this commentary. My views on the ontology of the world and the reasons on which I base my support for a systemic form of a plural materialism of spacetime and quantum fields can be found in the study by Romero (2018) and especially in Romero (2022).
